# Female Mice Deficient in Alpha-Fetoprotein Show Female-Typical Neural Responses to Conspecific-Derived Pheromones

**DOI:** 10.1371/journal.pone.0039204

**Published:** 2012-06-15

**Authors:** Olivier Brock, Matthieu Keller, Quentin Douhard, Julie Bakker

**Affiliations:** 1 Neuroendocrinology, Netherlands Institute for Neuroscience, Amsterdam, The Netherlands; 2 Behavioural and Reproductive Physiology, INRA/CNRS/University of Tours, Nouzilly, France; 3 Behavioral Neuroendocrinology, GIGA-Neurosciences, University of Liege, Liege, Belgium; 4 Medical Psychology, Vrije Universiteit Medical Center, Amsterdam, The Netherlands; Universitat de Barcelona, Spain

## Abstract

The neural mechanisms controlling sexual behavior are sexually differentiated by the perinatal actions of sex steroid hormones. We recently observed using female mice deficient in alpha-fetoprotein (AFP-KO) and which lack the protective actions of AFP against maternal estradiol, that exposure to prenatal estradiol completely defeminized the potential to show lordosis behavior in adulthood. Furthermore, AFP-KO females failed to show any male-directed mate preferences following treatment with estradiol and progesterone, indicating a reduced sexual motivation to seek out the male. In the present study, we asked whether neural responses to male- and female-derived odors are also affected in AFP-KO female mice. Therefore, we compared patterns of Fos, the protein product of the immediate early gene, *c-fos*, commonly used as a marker of neuronal activation, between wild-type (WT) and AFP-KO female mice following exposure to male or estrous female urine. We also tested WT males to confirm the previously observed sex differences in neural responses to male urinary odors. Interestingly, AFP-KO females showed normal, female-like Fos responses, i.e. exposure to urinary odors from male but not estrous female mice induced equivalent levels of Fos protein in the accessory olfactory pathways (e.g. the medial part of the preoptic nucleus, the bed nucleus of the stria terminalis, the amygdala, and the lateral part of the ventromedial hypothalamic nucleus) as well as in the main olfactory pathways (e.g. the piriform cortex and the anterior cortical amygdaloid nucleus), as WT females. By contrast, WT males did not show any significant induction of Fos protein in these brain areas upon exposure to either male or estrous female urinary odors. These results thus suggest that prenatal estradiol is not involved in the sexual differentiation of neural Fos responses to male-derived odors.

## Introduction

In mice, body odors provide essential information about the sex, social, and reproductive status of conspecifics [1] and may thus play a key role in mate recognition and mate preferences. These socially relevant odors are detected by either the main or the accessory olfactory system or both. The main olfactory system is usually used to detect volatile odors derived from food, predators and potential mates [2], whereas the accessory olfactory system is thought to detect non-volatile odors that influence reproductive and aggressive behaviors [3]. The accessory olfactory system has sexually dimorphic characteristics (morphological and functional) along its projection pathway, indicating an important role for sex steroid hormones in its development and functioning [4], [5], [6]. For instance, sex differences in immediate early gene (*c-Fos*) responses were observed along the entire accessory olfactory projection pathway when mice were exposed to bedding soiled by gonadally intact males [7]. These sex differences may reflect the perinatal action of estradiol in the male brain as male rats treated neonatally with an aromatase inhibitor (ATD: 1,4,6-androstatrien-3,17-dione) showed female-typical *c-Fos* responses when exposed to male odors [4]. However, using the aromatase knock-out mouse model (ArKO) which carries a targeted mutation in the aromatase gene thereby rendering these animals incapable of converting androgens into estrogens, Pierman et al [8] showed that male ArKO mice did not show female-typical neural Fos responses to male odors. This suggests that estradiol might not be involved in the sexual differentiation of olfactory responses in the mouse. Accordingly, Bodo & Rissman [9] showed that male Tfm mice (carrying the testicular feminization mutation of the androgen receptor), like WT females, showed Fos responses to male urinary odors in the medial preoptic area (MPOA) and in the bed nucleus of the stria terminalis (BnST), whereas no such induction was observed in males. This suggests that in contrast with the male rat, the sexual differentiation of neural *c-Fos* responses to male odors may not reflect the perinatal actions of estradiol, but those of androgens in the male mouse nervous system.

Mate preferences are controlled by neural mechanisms that are sexually differentiated by the perinatal actions of sex steroid hormones [10]. Interestingly, we recently observed that female mice carrying a mutation in the *Afp* gene (AFP-KO) which encodes the major fetal plasma protein alpha-fetoprotein that binds estradiol with high affinity did not show any male-directed mate preferences when tested under estrous conditions [11]. This finding is in line with our previous observations of female AFP-KO mice being clearly defeminized with regards to their female sexual behavior, i.e. lordosis behavior [12] as well as their GnRH/kisspeptin system, i.e. no steroid induced LH surges [13], [14]. As olfaction is essential for both mate recognition and the expression of courtship behaviors in mice [15], , we hypothesized here that the absence of male-directed mate preferences in AFP-KO females might reflect an inability to respond to male-derived olfactory cues. However, if true, this inability might reflect probably the integration of olfactory cues rather than their detection since we recently showed that AFP-KO animals can discriminate between male and female urinary odors [19].

Therefore, in the present study, we compared profiles of Fos protein between female WT and AFP-KO mice following exposure to male or estrous female urinary odors. We also included WT males as experimental group in order to confirm previously observed sex differences in neural Fos responses to male-, but not estrous female-, derived odors [7], [8].

## Results

### Accessory olfactory pathway

**Figure 1:**
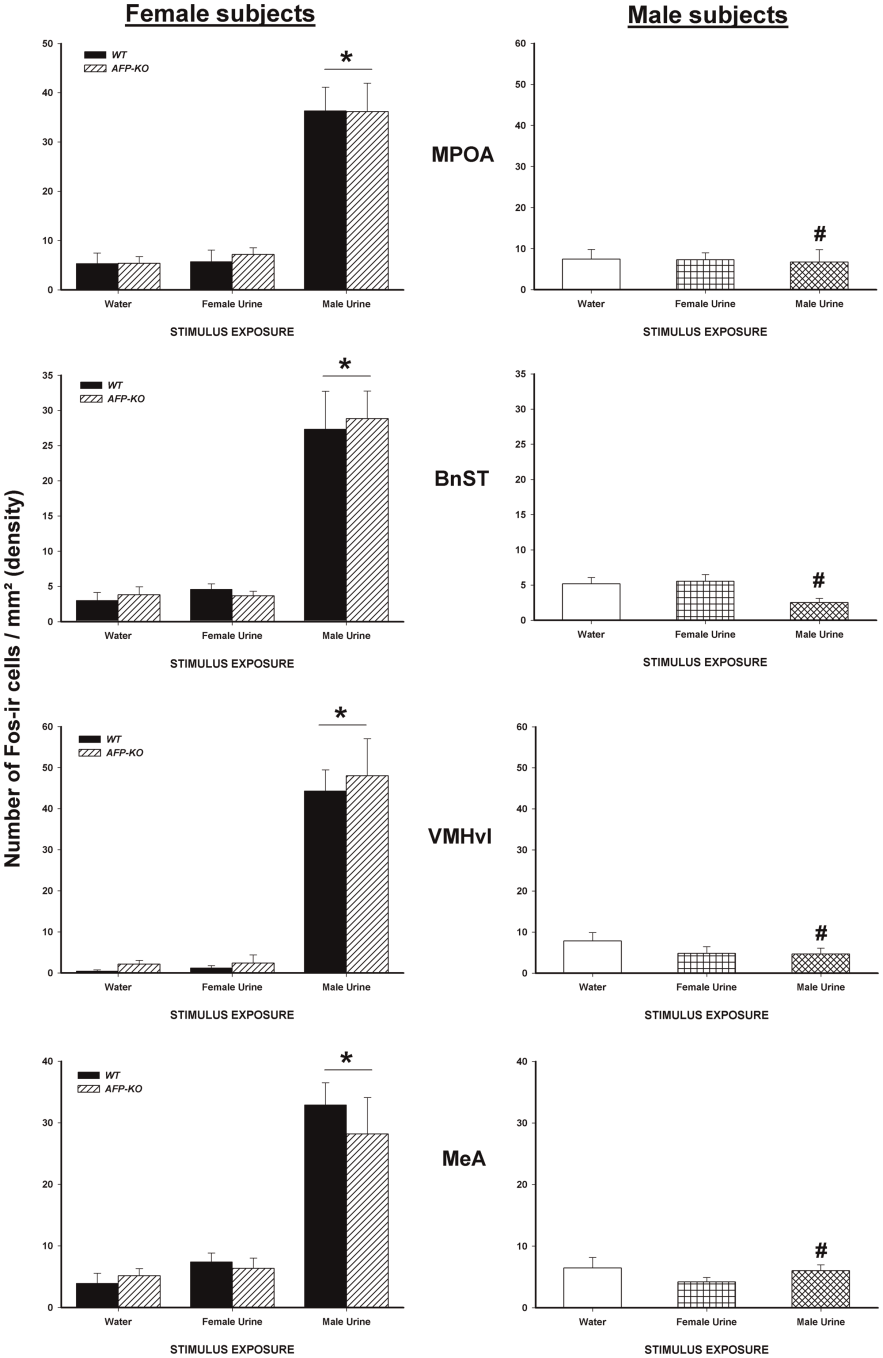
Exposure to male urinary odors increases the number of Fos-ir cells in WT and AFP-KO females but not in WT males. Mean +/− SEM number of Fos-ir cells per mm^2^ (density) in several brain regions which are part of the accessory olfactory pathway in male (intact) and female WT and AFP-KO female (ovariectomized and implanted with an E_2_ capsule) mice exposed to either water, estrous female or male urinary odors. * *P*<0.05 different from females that were exposed to water or to estrous female urine. # P<0.05 different from females of the same genotype exposed to intact male urine.

Overall, in WT females as well as in AFP-KO females, exposure to male urine, but not to estrous female urine, induced a significant expression of Fos in several brain regions receiving inputs from the accessory olfactory bulbs, including parts of the amygdala (MeA, MePV, MePD), the MPOA, the BnST and the VMH-vl (Fig. 1). These neural Fos responses to intact male urine were sexually dimorphic for all analyzed regions of the accessory olfactory pathways with only females (WT and AFP-KO), but not males, showing significant Fos activation. Exposure to estrous female urine did not lead to any significant Fos activation in either females (WT and AFP-KO) or males.

**Figure 2:**
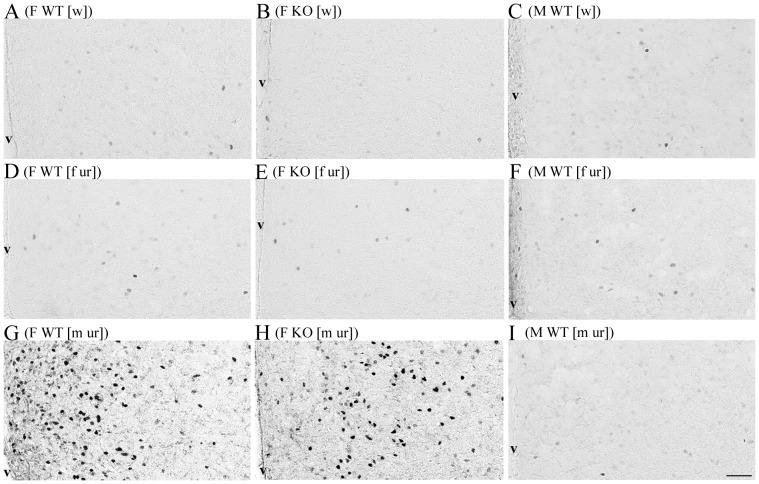
Fos induction in the MPOA following exposure to pheromones. Representative photomicrographs showing coronal sections of the MPOA with Fos-ir cells in a WT female (A, D, G), an AFP-KO female (B, E, H) and a WT male (C, F, I) following exposure to either deionized water (A, B, C), estrous female urine (D, E, F) or male urine (G, H, I). V, third ventricle. Scale bar, 600 µm.

ANOVA on the number of Fos-ir cells showed a significant interaction between sex, genotype and odor exposure in the MeA (F(4,63) = 13.38, *P*<0.001), MePD (F(4,63) = 9.64, *P*<0.001), MePV (F(4,63) = 6.16, *P*<0.001), MPOA (F(4,63) = 12.75, *P*<0.001), BnST (F(4,63) = 14.98, *P*<0.001) and the VMH-vl (F(4,63) = 16.88, *P*<0.001). In all of these brain regions, *post hoc* analysis indicated that females (WT and AFP-KO) showed significantly more Fos-ir cells when exposed to intact male urine, but not to estrous female urine, compared to females (WT and AFP-KO) exposed to deionized water. Furthermore, no significant Fos reponses were observed in any of the WT male groups (male urine *versus* estrous female urine *versus* deionized water; Fig. 2).

### Main olfactory pathway

**Figure 3:**
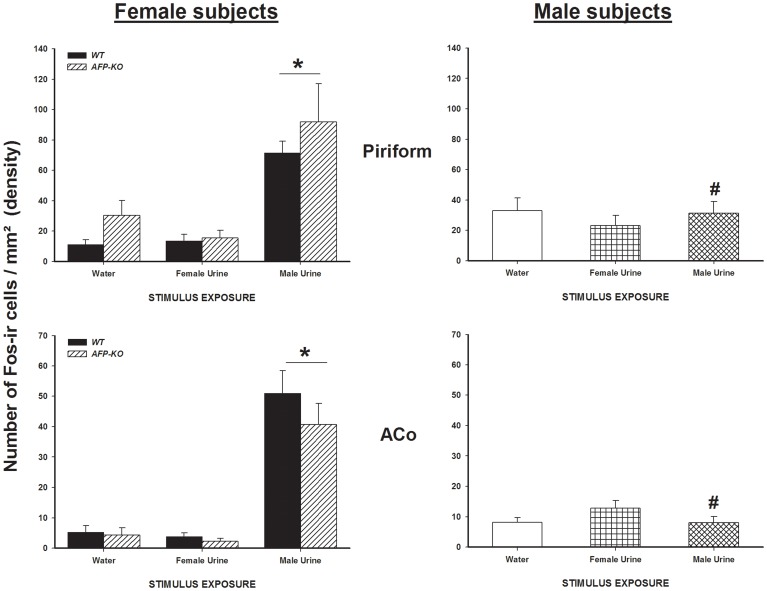
Exposure to male urinary odors increases the number of Fos-ir cells in WT and AFP-KO females but not in WT males. Mean +/− SEM number of Fos-ir cells per mm^2^ (density) in brain regions which are part of the main olfactory pathway in male (intact) and female WT and AFP-KO female (ovariectomized and implanted with an E_2_ capsule) mice exposed to either water, estrous female or male urinary odors. * *P*<0.05 different from females that were exposed to water or to estrous female urine. # P<0.05 different from females of the same genotype exposed to intact male urine.

Exposure to urine derived from intact males, but not from estrous females, induced significantly more Fos-ir cells in the piriform cortex and the ACo in WT and AFP-KO females than exposure to deionized water whereas WT males showed very few Fos-ir cells in response to either male or estrous female urine or deionized water (Fig. 3).

ANOVA on the number of Fos-ir cells showed a significant interaction between sex, genotype and odor exposure in the piriform cortex (F(4,63) = 8.73, *P*<0.001) and the ACo (F(4,63) = 10.87, *P*<0.001). *Post hoc* analysis indicated that the number of Fos-ir cells was significantly higher in WT and AFP-KO females when exposed to intact male urine, but not to estrous female urine, compared to deionized water, whereas WT males had equally low numbers of Fos-ir cells under all experimental conditions (male urine *versus* estrous female urine *versus* deionized water).

## Discussion

The present study showed that female mice carrying a targeted mutation in the *Afp* gene, encoding the important fetal plasma protein alpha-foetoprotein (AFP) that has high estradiol binding capacities [20], had normal, female-typical Fos responses to male and estrous female urinary odors in the main and accessory olfactory pathways despite the fact that they were exposed *in utero* to increased levels of estradiol. Male odors activated the BnST, the MPOA, the MeA and the VMH-vl which are target sites of olfactory inputs and play an important role in female reproductive behavior including olfactory preferences [21], [22]. This is in contrast with our previous finding [11] of no clear male-directed preferences in AFP-KO females when given the choice between an intact male and an estrous female, whereas WT females clearly preferred the intact male over the estrous female (see further discussion below). Furthermore, exposure to urinary odors derived from estrous females did not lead to a significant Fos activation in either experimental group (WT males, or WT and AFP-KO females). Thus, prenatal exposure to estradiol does not seem to influence neural Fos responses to urinary odors in the main and accessory olfactory systems in female mice. This result is surprising in light of our earlier studies showing a strong defeminizing role for prenatal estradiol in mate preferences and lordosis behavior [11], [12].

In the present study, we did not analyze neural Fos responses in the olfactory bulbs because it has already been shown that processing of sexually relevant odors by the olfactory bulbs was not affected by the hormonal status of the respondent [23]. In addition exposure to male- or estrous female-derived odors induced equivalent responses in Fos expression at the level of the main olfactory bulb (MOB) and the accessory olfactory bulb (AOB) in male and female mice [7] as well as in ArKO versus WT mice [8]. These studies thus suggest that the olfactory bulbs are probably not a critical target of estradiol action during perinatal development. The present study also confirms the presence of sexually dimorphic Fos responses in the accessory olfactory projection pathway to male urinary odors in WT mice as previously described by Halem [7], [24]. Sex differences were present in the main olfactory pathway (piriform cortex and ACo) and in the different parts of the amygdala (MeA, MePV, MePD), the MPOA, the BnST and the VMH-vl where neuronal Fos expression induced by male odors was high in female mice and completely absent in male mice. The female-typical Fos induction shown here in WT and AFP-KO female mice is in line with earlier studies by Bodo & Rissman [9] with regard to the MPOA and by Pierman et al [8] with regard to the VMH-vl for WT subjects. These two studies also showed that ArKO males have a normal male-typical Fos pattern, i.e. no Fos induction in the MPOA, and that Tfm males (carrying a targeted mutation of the androgen receptor) showed female-like Fos responses to male odors suggesting that estradiol was not involved in the sexual differentiation of neural Fos responses. The present study further confirms this hypothesis which is in sharp contrast with rat studies showing that perinatal action of estradiol in the male rat nervous system may influence this sexual differentiation; male rats treated with the aromatase inhibitor ATD showed a significant Fos response (female-like) in the MPOA following exposure to male bedding [4]. Furthermore, Bodo & Rissman [25] showed that by treating female mice on the day of birth with dihydrostestosterone (which is not aromatizable) Fos immunoreactivity was reduced in these females in the MPOA and in the BnST following exposure to male-soiled bedding.

In WT males, we observed very weak Fos responses in both the main and accessory olfactory pathway after exposure to either male or estrous female urinary odors. Our results confirm the absence of Fos activation in central brain regions observed after exposure to estrous female urine in WT and ArKO males [8]. However, several studies show that WT males can express significant Fos or MAPK responses in different hypothalamic regions after exposure to estrous female urine [26], [27] or to soiled bedding derived from estrous females [28],[29]. These discrepancies might be explained by the fact that the male subjects in our present study were sexually naive whereas the males in the other studies [26], [27], [28], [29] were sexually experienced before being exposed to female pheromones. It has been clearly shown in male rats that sexual experience with a receptive female may influence the induction of neural activation of central brain regions following exposure to urinary odors of a potential mate [30]. Furthermore, differences in odor composition between urine and soiled bedding might explain why some authors found a significant Fos [31] or MAPK [32] activation in the MeA, the MPOA and the VMHvl of sexually naïve WT males whereas we do not find such an activation following exposure to urinary odors.

As previously reported, female AFP-KO mice did not show any male-directed mate preferences [11]. The present study, however, shows that they had normal, female-typical, neural Fos responses in the accessory olfactory system when exposed to male- or estrous female-derived odors indicating the absence of a direct relationship between mate/odor preferences and neural Fos responses. A similar absence of a direct relationship has been described by Wersinger & Rissman [31]. They observed that male ERαKO mice (which lack a functional estrogen receptor α gene) showed no preferences when offered the choice between an estrous female and an intact male with direct access; however, these ERαKO males showed male-like Fos responses to female-soiled bedding suggesting that the neural substrates underlying the detection and processing of chemosensory cues could be uncoupled from those controlling mate preferences in mice. The same phenomenon has also been observed in rats; Bakker et al [4], [33] showed that unlike untreated littermates, male rats treated neonatally with ATD, failed to show a preference for a compartment with female-soiled bedding over a compartment with male-soiled bedding. Despite this lack of preference, the pattern of Fos-ir cells after exposure to female chemosensory cues was equivalent between untreated and ATD-treated male rats. All these data thus suggest that the expression of immediate early genes like *c-fos* in the brain in response to olfactory cues can be primarily used to determine whether an animal correctly perceives a particular olfactory stimulus and most likely not to predict any behavioral outcome, such as mate preferences. Thus, olfactory cues seemed to be correctly perceived by AFP-KO females [19] but perhaps they are not further processed by the neuroendocrine system, such as the kisspeptin/GnRH system, ultimately leading to the correct behavioral response. Female AFP-KO mice do not show any steroid induced luteinizing hormone surges most likely due to having only few kisspeptin neurons in the rostral periventricular area of the third ventricle [13], [14]. Some indirect support for this hypothesis comes from our observation [34] that kisspeptin neurons were activated by male urinary odors in female but not in male WT mice, and that male and female GPR54-KO mice (which lack the G-protein-coupled receptor that binds kisspeptin) failed to show any sexual partner preference in adulthood [35]. Thus AFP-KO females might lack the neural pathway that links the perception of an odor to its behavioral outcome, i.e. the appropriate male-directed preference.

## Materials and Methods

### Ethics statement

All experiments were conducted in accordance with the guidelines set forth by the National Institutes of Health “Guide for the Care and Use of Research Animals, Eight Edition”, and were approved by the Ethical Committees for Animal Use of the University of Liege (file number 525).

### Animals and hormonal treatment

All breeding and genotyping were performed at the GIGA Neurosciences, University of Liège, Liège, Belgium. In the present study, male and female mice heterozygous for the allele Afp^tm1lbmm^ (in the *CD1* background strain [12], [36]) were bred to generate wild type (WT) and homozygous-null (AFP-KO) offspring. Mice were genotyped by PCR analysis of tail DNA (for more detailed description, see [12], [37]). Subjects were weaned at 21 days and housed in same-sex groups, but mixed genotypes, under a reversed light-dark cycle (12 h∶ 12 h light/dark; 20.00 h lights on and 8.00 h lights off) in special light and temperature controlled housing units. Food and water were always available *ad libitum*. Three weeks before the stimulus exposure, male and female mice were placed into individual cages. Female mice (5 months of age) were ovariectomized under general anaesthesia after an intraperitoneal injection (i.p.) of a mixture of ketamine (80 mg/kg per mouse) and medetomidine (Domitor, Pfizer, 1 mg/kg per mouse). Mice received atipamezole (Antisedan, Pfizer, 4 mg/kg per mouse) subcutaneously at the end of the surgery in order to antagonize medetomidine-induced effects, thereby accelerating their recovery. During ovariectomy, females also received s.c. in the neck a 5-mm-long Silastic capsule (inner diameter: 1.57 mm; outer diameter: 2.41 mm) containing crystalline 17β-estradiol (diluted 1∶1 with cholesterol). The dose of E_2_ (E8875, Sigma) was based on a previous study (for details, see Bakker et al., 2002) showing that this treatment leads to estrous levels of estradiol. Males (5 months of age) were left gonadally intact. Two weeks after surgery, animals were randomized to three stimulus exposure groups: male urine (WT: 9 males and 9 females; AFP-KO: 6 females), estrous female urine (WT: 9 males and 8 females; AFP-KO: 8 females) and deionized water to serve as control (WT: 8 males and 8 females; AFP-KO: 7 females).

### Urine collection

Male urine was collected from 8 gonadally intact *CD1* males by holding the mouse by the scruff of the neck over an Eppendorf vial and by pushing gently on its bladder. Female urine was collected from 6 ovariectomized/E_2_ implanted CD1 females; four hours before urine collection, these females received a s.c. injection of progesterone (500 µg) to induce a proestrus condition. Same-sex urine stimulus samples were pooled and subsequently aliquoted in 500 µl Eppendorf vials and stored at −80°C until use.

### Urine exposure

Male and female subjects were housed in two separate housing units to minimize their exposure to opposite-sex odors. Subjects were then trained daily, during 5 days, to the manipulation used for urine exposure. During the dark phase of the light/dark cycle, animals were taken out of their home cage and received 30 µl of deionized water directly on the top of their nose and were then placed back into their home cage. On the day of testing, mice were exposed to either intact male urine, estrous female urine or to deionized water to serve as control. The three groups were housed separately in ventilated housing units. After exposure to the odor stimulus, the subject was placed back again into its home cage. We chose to apply the urine directly onto the nose instead of giving free access to the urine since previous studies showed that applying urine onto the nose directly activated both the main and the accessory olfactory system [8], [26], [27], [38], [39]. Furthermore, we wanted to exclude any confounding effects of differences in olfactory investigation of the urinary odors by the different experimental groups.

Ninety minutes following the initial urine exposure, subjects were anaesthetized and perfused transcardially with saline followed immediately by 4% cold paraformaldehyde. Brains were removed and postfixed in 4% paraformaldehyde for 2 hours. Then brains were cryoprotected in 30% sucrose/PBS solution and when sunken, frozen on dry ice and stored at −80°C. Sections of 30 µm were cut on a Leica CM3050S cryostat. Forebrains were cut coronally from the level of the nucleus accumbens until the end of the hippocampus. Sections were saved in four different series, placed in antifreeze solution, and stored at −20°C for later immunocytochemistry.

### Immunocytochemistry

All brain sections were processed for Fos-immunoreactivity as previously described [8], [24]. All incubations were carried out at room temperature (22°C) and all washes of brain tissue sections were performed using tris-buffered saline (TBS) or tris-buffered saline containing 0.1% Triton X-100 (TBST). Briefly, endogenous peroxidase activity was quenched by incubating the sections for 30 min with 3% hydrogen peroxide. Aspecific binding sites were then blocked by incubating sections for 1 h with 5% normal goat serum (Dako Cytomation, Denmark). Sections were then incubated overnight with a rabbit polyclonal anti-*c-fos* antibody (1∶2000 in TBST/5% NGS; Santa Cruz – c-Fos (4): sc-52R) followed by an incubation for 1 h in a goat anti-rabbit biotinylated antibody (1∶1000 in TBST; Dako Cytomation, Denmark). Sections were then incubated for 1 h in avidin-biotin complex (1/800, ABC, Vector Laboratory, Burlingame, CA) and then reacted for 8 min with 3,3′diaminobenzidine tetrahydrochloride (DAB Kit, Vector Laboratory). Sections were then washed, dried overnight, left in xylene (Sigma) for 15 min and coverslipped using Eukit (Fluka, Steinheim, Germany).

### Data analysis

**Figure 4:**
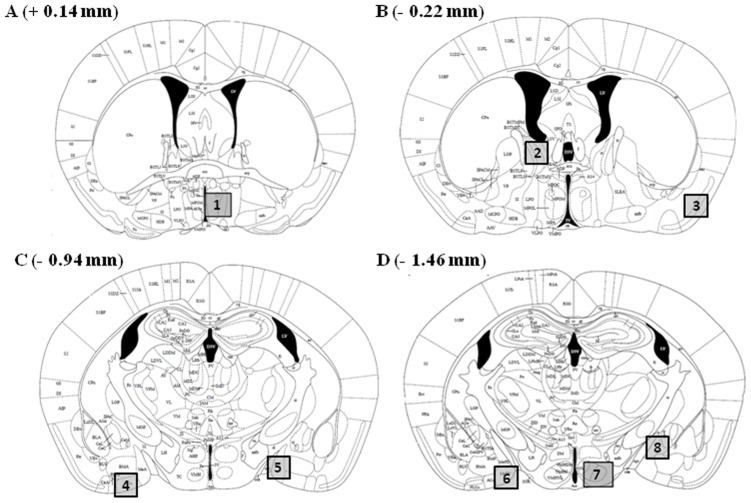
Drawings taken from the mouse brain atlas of Paxinos and Franklin [40] showing the location of forebrain regions where Fos-ir cells were quantified (*shaded areas* in each panel). The medial part of the medial preoptic nucleus [MPOA (A1: Figure 30 of the mouse atlas: interaural, 3.94 mm; bregma, 0.14 mm)], the bed nucleus of the stria terminalis [BnsT (B2: Figure 33 of the mouse atlas: interaural, 3.58 mm; bregma, −0.22 mm)], the piriform cortex [PIR (B3: Figure 33 of the mouse atlas: interaural, 3.58 mm; bregma, −0.22 mm)], the anterior cortical amygdaloid nucleus [ACo (C4: Figure 39 of the mouse atlas: interaural, 2.86 mm; bregma, −0.94 mm)], the medial amygdala [MeA (C5: Figure 39 of the mouse atlas: interaural, 2.86 mm; bregma, −0.94 mm)], the posteroventral part of the medial amygdala [MePV (D6: Figure 43 of the mouse atlas: interaural, 2.34 mm; bregma, −1.46 mm)], the ventrolateral part of the ventromedial hypothalamic nucleus [VMH-vl (D7: Figure 43 of the mouse atlas: interaural, 2.34 mm; bregma, −1.46 mm)] and the posterodorsal part of the medial amygdale [MePD (D8: Figure 43 of the mouse atlas: interaural, 2.34 mm; bregma, −1.46 mm)]. The distance of each coronal brain slice in front of (+) or behind (−) bregma is given for each panel.

Numbers of Fos-immunoreactive (Fos-ir) cells were counted unilaterally in one representative section of several brain nuclei (Fig. 4) implicated in the accessory and main olfactory pathways and thus in the control of sexual behavior [40]. Numbers of Fos-ir cells were quantified by an experimenter who was blind to the experimental treatment of mice. Brain sections were digitized through a video camera (Scion Corporation, CFW 1612C) attached to a microscope (Olympus MTV-3 – 20X objective), and immunoreactivity was quantified with a PC-based image analysis system using the particle-counting protocol of the NIH Image program (Version 1.37; Wayne Rasband, NIH, Bethesda, MD, USA). Digital images were made binary, and a manual threshold was used for discriminating the labelled material from the background. The number of Fos-ir cells was measured in one entire field, specifically defined for each brain region and placed in a standardized manner based on pre-defined anatomical landmarks in each single section. With a 20X objective, exclusion thresholds were set at 80 (low threshold) and 800 (high threshold) pixels to remove from the counts dark objects that were not the same size as a cell nucleus.

### Statistics

Numbers of Fos-ir cells were analyzed using two-way analysis of variance (ANOVA – Statistica 10.0) with the number of Fos-ir cells as the dependent measure, sex/genotype (3) and stimulus exposure (3) as independent factors. When appropriate, all ANOVAs were followed by Fisher Least Significant Difference *post-hoc* comparisons. Only significant (*P*<0.05) effects are presented above.

## References

[1] Brown RE (1979). Mammalian social odors: a critical review.. Adv Study Behav.

[2] Firestein S (2001). How the olfactory system makes sense of scents.. Nature.

[3] Keverne EB (1999). The vomeronasal organ.. Science.

[4] Bakker J, Baum MJ, Slob AK (1996). Neonatal inhibition of brain estrogen synthesis alters adult neural Fos responses to mating and pheromonal stimulation in the male rat.. Neuroscience.

[5] Bressler SC, Baum MJ (1996). Sex comparison of neuronal Fos immunoreactivity in the rat vomeronasal projection circuit after chemosensory stimulation.. Neuroscience.

[6] Guillamon A, Segovia S (1997). Sex differences in the vomeronasal system.. Brain Res Bull.

[7] Halem HA, Cherry JA, Baum MJ (1999). Vomeronasal neuroepithelium and forebrain Fos responses to male pheromones in male and female mice.. J Neurobiol.

[8] Pierman S, Douhard Q, Bakker J (2008). Evidence for a role of early oestrogens in the central processing of sexually relevant olfactory cues in female mice.. Eur J Neurosci.

[9] Bodo C, Rissman EF (2007). Androgen receptor is essential for sexual differentiation of responses to olfactory cues in mice.. Eur J Neurosci.

[10] Bakker J (2003). Sexual differentiation of the neuroendocrine mechanisms regulating mate recognition in mammals.. J Neuroendocrinol.

[11] Brock O, Bakker J (2011). Potential contribution of prenatal estrogens to the sexual differentiation of mate preferences in mice.. Horm Behav.

[12] Bakker J, De Mees C, Douhard Q, Balthazart J, Gabant P (2006). Alpha-fetoprotein protects the developing female mouse brain from masculinization and defeminization by estrogens.. Nat Neurosci.

[13] De Mees C, Laes JF, Bakker J, Smitz J, Hennuy B (2006). Alpha-fetoprotein controls female fertility and prenatal development of the gonadotropin-releasing hormone pathway through an antiestrogenic action.. Mol Cell Biol.

[14] Gonzalez-Martinez D, De Mees C, Douhard Q, Szpirer C, Bakker J (2008). Absence of gonadotropin-releasing hormone 1 and Kiss1 activation in alpha-fetoprotein knockout mice: prenatal estrogens defeminize the potential to show preovulatory luteinizing hormone surges.. Endocrinology.

[15] Edwards DA, Burge KG (1973). Olfactory control of the sexual behavior of male and female mice.. Physiol Behav.

[16] Keller M, Douhard Q, Baum MJ, Bakker J (2006). Destruction of the main olfactory epithelium reduces female sexual behavior and olfactory investigation in female mice.. Chem Senses.

[17] Keller M, Pierman S, Douhard Q, Baum MJ, Bakker J (2006). The vomeronasal organ is required for the expression of lordosis behaviour, but not sex discrimination in female mice.. Eur J Neurosci.

[18] Thompson ML, Edwards DA (1972). Olfactory bulb ablation and hormonally induced mating in spayed female mice.. Physiol Behav.

[19] Keller M, Pawluski JL, Brock O, Douhard Q, Bakker J (2010). The alpha-fetoprotein knock-out mouse model suggests that parental behavior is sexually differentiated under the influence of prenatal estradiol.. Horm Behav.

[20] Raynaud JP, Bouton MM, Gallet-Bourquin D, Philibert D, Tournemine C (1973). Comparative study of estrogen action.. Mol Pharmacol.

[21] Choi GB, Dong HW, Murphy AJ, Valenzuela DM, Yancopoulos GD (2005). Lhx6 delineates a pathway mediating innate reproductive behaviors from the amygdala to the hypothalamus.. Neuron.

[22] Robarts DW, Baum MJ (2007). Ventromedial hypothalamic nucleus lesions disrupt olfactory mate recognition and receptivity in female ferrets.. Horm Behav.

[23] Pfaff DW, Pfaffmann C (1969). Olfactory and hormonal influences on the basal forebrain of the male rat.. Brain Res.

[24] Halem HA, Baum MJ, Cherry JA (2001). Sex difference and steroid modulation of pheromone-induced immediate early genes in the two zones of the mouse accessory olfactory system.. J Neurosci.

[25] Bodo C, Rissman EF (2008). The androgen receptor is selectively involved in organization of sexually dimorphic social behaviors in mice.. Endocrinology.

[26] Reyes R, Mendoza J, Ballesteros J, Moffat C (2004). Males chemosignals inhibit the neural responses of male mice to female chemosignals.. Brain Res Bull.

[27] Swaney WT, Curley JP, Champagne FA, Keverne EB (2008). The paternally expressed gene Peg3 regulates sexual experience-dependent preferences for estrous odors.. Behav Neurosci.

[28] Aste N, Honda S, Harada N (2003). Forebrain Fos responses to reproductively related chemosensory cues in aromatase knockout mice.. Brain Res Bull.

[29] Taziaux M, Keller M, Balthazart J, Bakker J (2011). Rapid activation of phosphorylated mitogen-actived protein kinase after sexual motivation in male mice.. Neuroreport.

[30] Hosokawa N, Chiba A (2005). Effects of sexual experience on conspecific odor preference and estrous odor-induced activation of the vomeronasal projection pathway and the nucleus accumbens in male rats.. Brain Res.

[31] Wersinger SR, Rissman EF (2000). Oestrogen receptor alpha is essential for female-directed chemo-investigatory behaviour but is not required for the pheromone-induced luteinizing hormone surge in male mice.. J Neuroendocrinol.

[32] Dudley CA, Chakravarty S, Barnea A (2001). Female odors lead to rapid activation of mitogen-activated protein kinase (MAPK) in neurons of the vomeronasal system.. Brain Res.

[33] Bakker J, Van Ophemert J, Slob AK (1996). Sexual differentiation of odor and partner preference in the rat.. Physiol Behav.

[34] Bakker J, Pierman S, Gonzalez-Martinez D (2010). Effects of aromatase mutation (ArKO) on the sexual differentiation of kisspeptin neuronal numbers and their activation by same versus opposite sex urinary pheromones.. Horm Behav.

[35] Kauffman AS, Gottsch ML, Roa J, Byquist AC, Crown A (2007). Sexual differentiation of Kiss1 gene expression in the brain of the rat.. Endocrinology.

[36] Gabant P, Forrester L, Nichols J, Van Reeth T, De Mees C (2002). Alpha-fetoprotein, the major fetal serum protein, is not essential for embryonic development but is required for female fertility.. Proc Natl Acad Sci U S A.

[37] Bakker J, De Mees C, Szpirer J, Szpirer C, Balthazart J (2007). Exposure to oestrogen prenatally does not interfere with the normal female-typical development of odour preferences.. J Neuroendocrinol.

[38] Kang N, Janes A, Baum MJ, Cherry JA (2006). Sex difference in Fos induced by male urine in medial amygdala-projecting accessory olfactory bulb mitral cells of mice.. Neurosci Lett.

[39] Pankevich DE, Cherry JA, Baum MJ (2006). Accessory olfactory neural Fos responses to a conditioned environment are blocked in male mice by vomeronasal organ removal.. Physiol Behav.

[40] Paxinos G, Franklin KBJ (2001). The Mouse Brain in Stereotaxic Coordinates.. Academic Press, San Diego.

